# Lectin pathway components and autoantibodies as novel immunological biomarkers in systemic lupus erythematosus (SLE) patients from Western India

**DOI:** 10.1038/s41598-025-10891-5

**Published:** 2025-10-30

**Authors:** Kirti Rai, Ridi Khatri, Amrutha Jose, Harshada Konkar, Milind Nadkar, Anjali Rajadhyaksha, Lubka Roumenina, Altaf Parande, Gauthami Bitla, Vijay Padwal, Manisha Madkaikar, Vandana Pradhan

**Affiliations:** 1 Department of Clinical and Experimental Immunology, ICMR-National Institute of Immunohaematology, Mumbai, India; 2 ICMR-National Institute of Immunohaematology, Mumbai, India; 3 Department of Medicine, G.S. Medical College and King Edward Memorial Hospital, Mumbai, India; 4 Cordeliers Research Center, Inflammation Complement and Cancer, INSERM UMRS 1138, Paris, France; 5 Library and IT, ICMR-National Institute of Immunohaematology, Mumbai, India; 6 Department of Pediatric Immunology & Leukocyte Biology, ICMR- National Institute of Immunohaematology, Mumbai, India

**Keywords:** Systemic lupus erythematosus, Lectin pathway, Ficolins, MBL, Autoantibodies, Disease activity, Immunology, Rheumatology

## Abstract

The lectin pathway of complement aids in removing apoptotic cells and maintenance of tissue homeostasis. However, its role in SLE pathogenesis remains unknown. This study aimed to assess the association of ficolins, mannose-binding lectin (MBL), and other pathogen recognition molecules (PRMs) of the lectin pathway and their corresponding autoantibodies with various clinical manifestations and disease activity in SLE patients from Western India. In this cross-sectional study, 282 clinically diagnosed SLE patients were included. Serum levels of ficolins, antigenic MBL, MBL-associated serine proteases (MASPs), MBL-associated protein 44 (MAp44), Collectin liver-1 (CL-L1), and their corresponding autoantibodies were quantified using ELISA. Group differences were analyzed using Mann-Whitney U tests, while associations/relationships were evaluated using chi-square tests and Spearman’s correlations. Serum levels of ficolin-2 (*p < 0.001*), MASP-3 (*p = 0.030*), and MAp44 (*p < 0.001*) were significantly elevated, while antigenic MBL (*p < 0.001*) and MASP-1 (*p < 0.001*) were significantly reduced in SLE patients compared to healthy controls (HCs). Renal involvement was associated with elevated ficolin-1 (*p = 0.009*), while hematological manifestations were linked to reduced MASP-1 (*p = 0.018*), MASP-3 (*p = 0.002*), and MAp44 (*p = 0.002*) levels. Mucocutaneous manifestations were associated with elevated MAp44 (*p < 0.001*) and anti-ficolin-1 (*p = 0.038*) autoantibodies. Anti-ficolin-1 (*p = 0.001*), anti-ficolin-2 (*p = 0.001*), and anti-ficolin-3 (*p < 0.001*) autoantibodies were significantly elevated in SLE patients compared to HCs. Anti-ficolin-2 autoantibodies were negatively correlated with ficolin-2 (*r*=-0.153, *p* = 0.015). Anti-MBL antibodies were correlated with SLEDAI (*r* = 0.169, *p* = 0.007) and anti-dsDNA antibodies (*r* = 0.178, *p* = 0.005). These findings indicate altered levels of lectin pathway-associated PRMs and their corresponding autoantibodies in SLE. Their association with clinical manifestations, disease activity, and complement-related parameters, suggest their potential as novel biomarkers in SLE.

## Introduction

Systemic Lupus Erythematosus (SLE) is a complex autoimmune disease characterized by a wide range of clinical manifestations. A complex interaction of genetic, environmental, and immunological factors contributes to SLE pathogenesis^1^. The complement is an important component of the innate immunity and is involved in SLE pathogenesis. There are three types of complement activation pathways: classical, alternative, and lectin^2^.

The lectin pathway is an antibody-independent complement activation pathway. Collectins, ficolins, and mannose-binding lectin (MBL) are the pattern recognition molecules (PRMs) of the lectin pathway. There are three types of ficolins, namely, ficolin-1 (M-ficolin), ficolin-2 (L-ficolin), and ficolin-3 (H-ficolin), in humans, which are associated with MBL-associated serine proteases (MASPs), having effector functions and MBL-associated proteins (MAps), having regulatory functions in lectin pathway^3^. The binding of characteristic glycan structures in pathogen cell walls on PRMs initiates the lectin pathway activation that is essential for the removal of apoptotic cells and the maintenance of tissue homeostasis^4–6^.

Previous studies have demonstrated that the lectin pathway is dysregulated in SLE and is involved in its pathogenesis^3,6,7^. Additionally, polymorphisms in genes encoding these PRMs of the lectin pathway are associated with their altered levels and have been implicated in SLE susceptibility^8–10^. The involvement of the complement in SLE pathogenesis is further supported by the presence of antibodies targeting various complement proteins. However, studies assessing autoantibodies against various components of the lectin pathway are limited, and their significance remains largely unexplored in the available literature. Based on this, we hypothesized that serum levels of lectin pathway PRMs and their corresponding autoantibodies are associated with clinical manifestations and disease activity in SLE. The present study on SLE patients from Western India aimed to measure the serum levels of lectin pathway–associated PRMs and their corresponding autoantibodies, and to assess their associations with clinical manifestations, disease activity, and complement-related parameters.

## Methods

### Patients and controls

A total of 282 SLE patients who were referred to ICMR-NIIH in Mumbai, India, over a 2- year period (2021–2023), were enrolled in this cross-sectional study. The American College of Rheumatology (ACR) criteria was used for SLE diagnosis^11^. The study was conducted in adherence to the Declaration of Helsinki. The ethical approval was received from the Institutional Ethics Committee for Research on Human Subjects, ICMR–National Institute of Immunohaematology (ICMR-NIIH), Mumbai, India (ICMR-NIIH/IEC/07/2020). Written informed consent was taken from all participants prior to enrollment. Demographic details and clinical manifestations were recorded in the case record forms. The Safety of Estrogens in Lupus Erythematosus National Assessment-Systemic Lupus Erythematosus Disease Activity Index (SELENA-SLEDAI) score was used to evaluate the disease activity^12^. Patients with hypertension, diabetes, significant hyperlipidemia, pregnant, post-menopausal women, and smokers were not included in this study. Sample sera were stored at -80℃ until tested. For comparison, serum samples were also obtained from 50 age- and sex-matched healthy controls (HCs), after taking their consent.

### Assessment of serum levels of lectin pathway-associated PRMs and other serological parameters

Serum concentrations of ficolin-1, ficolin-2, ficolin-3, and antigenic MBL levels were measured in 282 SLE patients using commercially available ELISA kits (HK357, HK336, HK340, and HK323 respectively, Hycult Biotech, Netherlands). MASP-1 (MBS764590) and MAp44 (MBS1607689) serum levels were determined using ELISA kits from My BioSource. MASP-3 (SB-EKH4912) and CL-L1 (SB-EKH4136) serum levels were determined using ELISA kits from SARD Biosciences, MH, India. Circulating immune complexes (C1q-CIC) levels were estimated using the C1q-CIC ELISA kit (EIA-3169, DRG International, USA). Levels of high sensitivity C-Reactive protein (hsCRP), along with complement components C3 and C4, were determined using MISPA-i3 Nephelometer (Agappe, Kerala, India).

### Assessment of autoantibodies against Ficolins and MBL

The autoantibodies were assessed in a subset of 250 SLE patients due to limited sample availability from the total cohort of 282 patients. Recombinant proteins were obtained from R&D Systems, Inc., USA, for ficolin-1; Cusabio Biotech Co., TX, USA, for ficolin-2 and MBL; and My BioSource for ficolin-3 (MBS2009080). The method outlined by Colliard et al. was used to detect anti-ficolin (anti-ficolin-1, anti-ficolin-2, anti-ficolin-3) autoantibodies^5^, and anti-MBL antibodies were assessed using the method described by Takahashi et al.^13^, with minor modifications. Briefly, recombinant ficolin-1, ficolin-2, ficolin-3, and MBL protein were coated into high-binding 96-well microtiter plates at a final concentration of 1 µg/ml in 0.5 M carbonate–bicarbonate buffer (15mM Na_2_CO_3_, 35mM NaHCO_3_, pH 9.6), and incubated overnight at 4 °C. After coating, plate was washed with phosphate-buffered saline (PBS) containing 0.05% Tween-20 (PBS-T) and blocked with 1% bovine serum albumin (BSA) in PBS for 1 h at 37 °C. Serum samples, diluted 1:50 in PBS-T supplemented with 1% BSA, were added to each well and incubated at 37 °C for one hour. Following washing, bound antibodies were detected using goat anti-human IgG conjugated to horseradish peroxidase (HRP) (1:80000 diluted in PBS with 1% BSA) for anti-ficolin assays and to alkaline phosphatase (1:5000 diluted in PBS-T) for anti-MBL assay for one hour at 37 °C. For colour development, TMB substrate was added for anti-ficolin assays and p-nitrophenyl phosphate (2 mg/ml diluted in diluted in 0.5 M carbonate-bicarbonate buffer) for anti-MBL assay. The reaction was stopped with 0.5M H_2_SO_4_ for anti-ficolins and 4 N NaOH for anti-MBL assay. Absorbance was measured at 405 nm. Inhibition assays were used to validate the presence of anti-MBL antibodies. Recombinant MBL with different concentrations (5, 10, and 1 gm/mL) were added to anti-MBL positive sera. The criteria for positivity were established based on the 98th percentile of the OD readings from the control group, with a cut-off value set at 95 arbitrary units (AU). Autoantibody levels were expressed in AU, calculated by normalizing the OD of each sample to the OD of a positive control (pooled sera from five SLE patients previously confirmed to have high autoantibody levels), which was arbitrarily assigned a value of 100 AU. The formula used was: $AU = {\text{ }}\left( {OD_{{sample}} /OD_{{positive{\text{ }}control}} } \right){\text{ }} \times {\text{ }}100$

Anti-dsDNA antibodies were determined by a commercially available indirect ELISA kit (EUROIMMUN, Germany) and anti-C1q antibodies were assessed using an anti-C1q ELISA kit (EIA5762, DRG International, USA).

### Statistical analysis

Statistical analysis was carried out using IBM SPSS STATISTICS 27 and R (version 4.4.2). The normality of continuous variables was assessed using the Kolmogorov–Smirnov and Shapiro-Wilk test. As most variables did not follow a Gaussian distribution, non-parametric tests were applied. The categorial data are represented as frequency (%) and continuous variables are represented with medians, along with the first (Q_1_) and third quartiles (Q_3_); the interquartile range (IQR) is defined as Q_3_-Q_1_. The Mann-Whitney U test was applied to compare continuous variables between groups, and associations between categorical variables were evaluated using the chi-square test. Correlations between the variables were assessed using Spearman’s rank correlation. A p-value of less than 0.05 was considered statistically significant.

## Results

### Patient demographics and clinical characteristics

Summarizes the demographic and clinical characteristics of the 282 SLE patients included in this study. The median (Q_1_, Q_3_) age at the time of enrolment was 30 (22, 40) years for SLE patients with 88.3% females. Based on the SLEDAI score, 144 patients (51.1%) were classified as active SLE, and 138 patients (48.9%) were classified as inactive SLE. Among clinical manifestations, renal involvement was the most prevalent, affecting 112 (39.7%) patients, followed by constitutional manifestations in 111 patients (39.4%). Hematological manifestations were observed in 96 patients (34.0%). ANA positivity was observed in majority of both inactive (95.7%) and active (96.5%) SLE patients. The median (Q_1_, Q_3_) levels of anti-dsDNA antibodies were elevated among active SLE patients: 321.83 iu/ml (51.37, 676.87) as compared to inactive SLE patients: 92.40 iu/ml (9.90, 480.26). Complement consumption was more prevalent among the active SLE patients, with 72.2% showing low antigenic C3 levels and 71.5% with low antigenic C4, as compared to 44.2% and 51.4%, respectively, in inactive SLE patients. Low antigenic C3 and C4 levels were also more frequently observed among active SLE patients (59.0%) than in inactive SLE patients (37.7%).

**Table 1:** Demographic, clinical and laboratory characteristics of SLE patients (*n* = 282).

Demographic characteristics		
Age at enrolment (years)	30 (22, 40)	
Age of onset (years)	27 (20, 36)	
Females	249 (88.3%)	
Males	33 (11.7%)	
Duration of treatment (months)	6 (1, 42)	
SLEDAI score	7 (4, 11)	
Inactive SLE (SLEDAI < 7)	138 (48.9%)	
Active SLE (SLEDAI ≥ 7)	144 (51.1%)	
Clinical manifestations		
Constitutional	111 (39.4%)	
Hematological	96 (34.0%)	
Neuropsychiatric	9 (3.2%)	
Mucocutaneous	56 (19.9%)	
Serositis (Pleuritis, Pericarditis)	8 (2.8%)	
Musculoskeletal	49 (17.4%)	
Renal	112 (39.7%)	

	Inactive SLE	Active SLE
Laboratory parameters		
ANA positivity	132 (95.7%)	139 (96.5%)
Anti-dsDNA antibody (IU/ml)	92.40 (9.90, 480.26)	321.83 (51.37, 676.87)
Low antigenic C3	61 (44.2%)	104 (72.2%)
Low antigenic C4	71 (51.4%)	103 (71.5%)
Low antigenic C3 and C4	52 (37.7%)	85 (59.0%)

Continuous variables are represented as median (Q_1_, Q_3_) and categorial data is represented as frequency (%).

### Serum levels of lectin pathway-associated PRMs in SLE patients in comparison to HCs

**Fig. 1:**
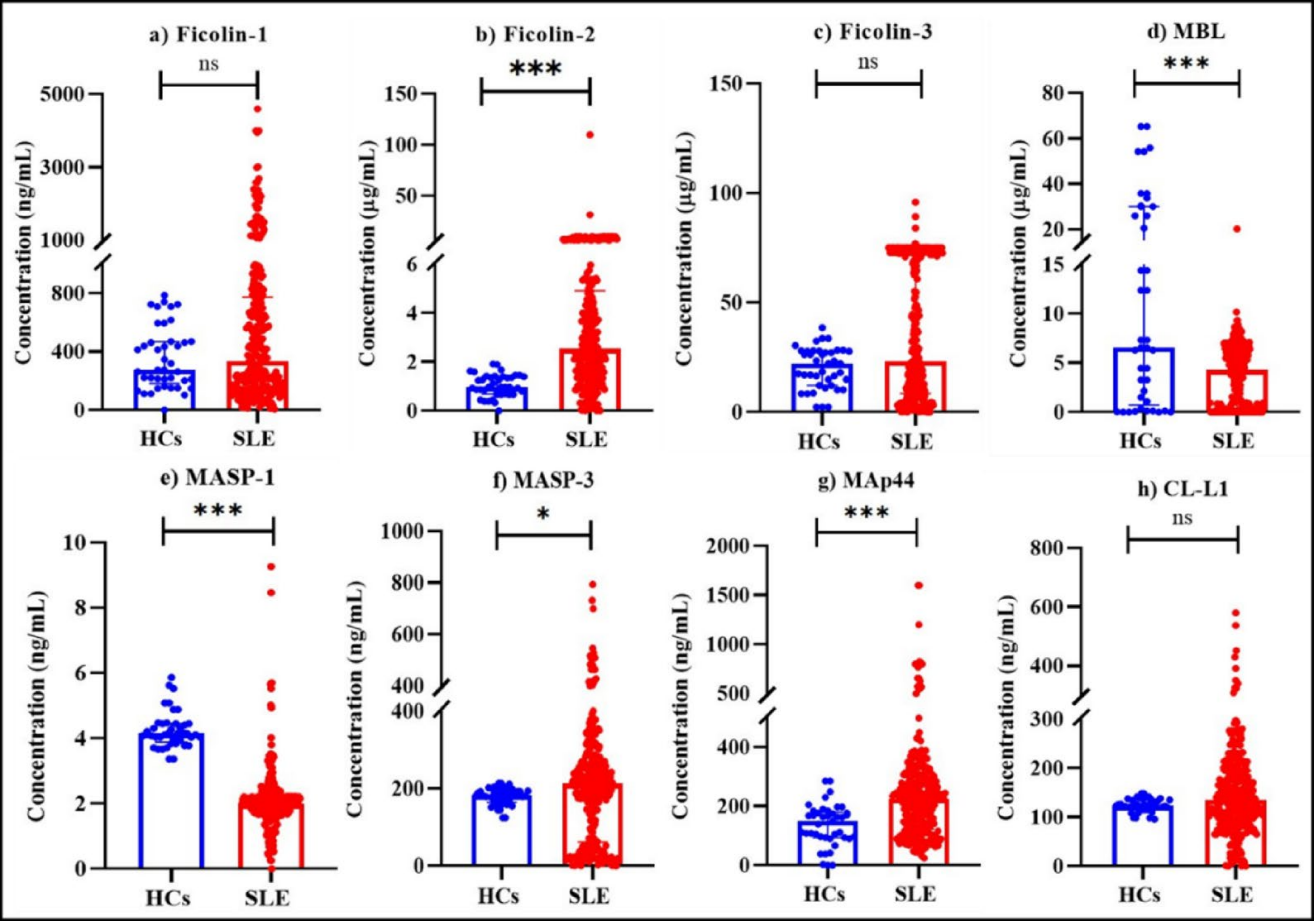
Comparison of serum levels of lectin pathway PRMs between HCs (*n* = 50) and SLE patients (*n* = 282). Serum levels of (**a**) ficolin-1, (**b**) ficolin-2, (**c**) ficolin-3, (**d**) MBL, (**e**) MASP-1, (**f**) MASP-3, (**g**) MAp44 and (**h**) CL-L1 were measured using ELISA. Data are presented as individual values (dots), with bars representing the medians. Group comparisons were performed using the Mann-Whitney U test. Statistical significance is indicated as follows: *p < 0.05, **p < 0.01, ***p < 0.001.

Alterations in the lectin pathway-associated PRMs were assessed by comparing their serum levels among SLE patients and HCs. It was observed that in comparison to HCs, SLE patients had elevated ficolin-2 (*p < 0.001*), MASP-3 (*p = 0.030*), and MAp44 (*p < 0.001*) levels (Fig. 1 (b), (f), (g)). Conversely, SLE patients had significantly reduced antigenic MBL (p < 0.001) and MASP-1 (*p < 0.001*) levels than HCs(Fig. 1 (d) and (e)). No significant differences were observed in ficolin-1, ficolin-3, and CL-L1 levels (Fig. 1 (a), (c) and (h)).

### Serum levels of lectin pathway-associated PRMs in active and inactive SLE patients

**Fig. 2:**
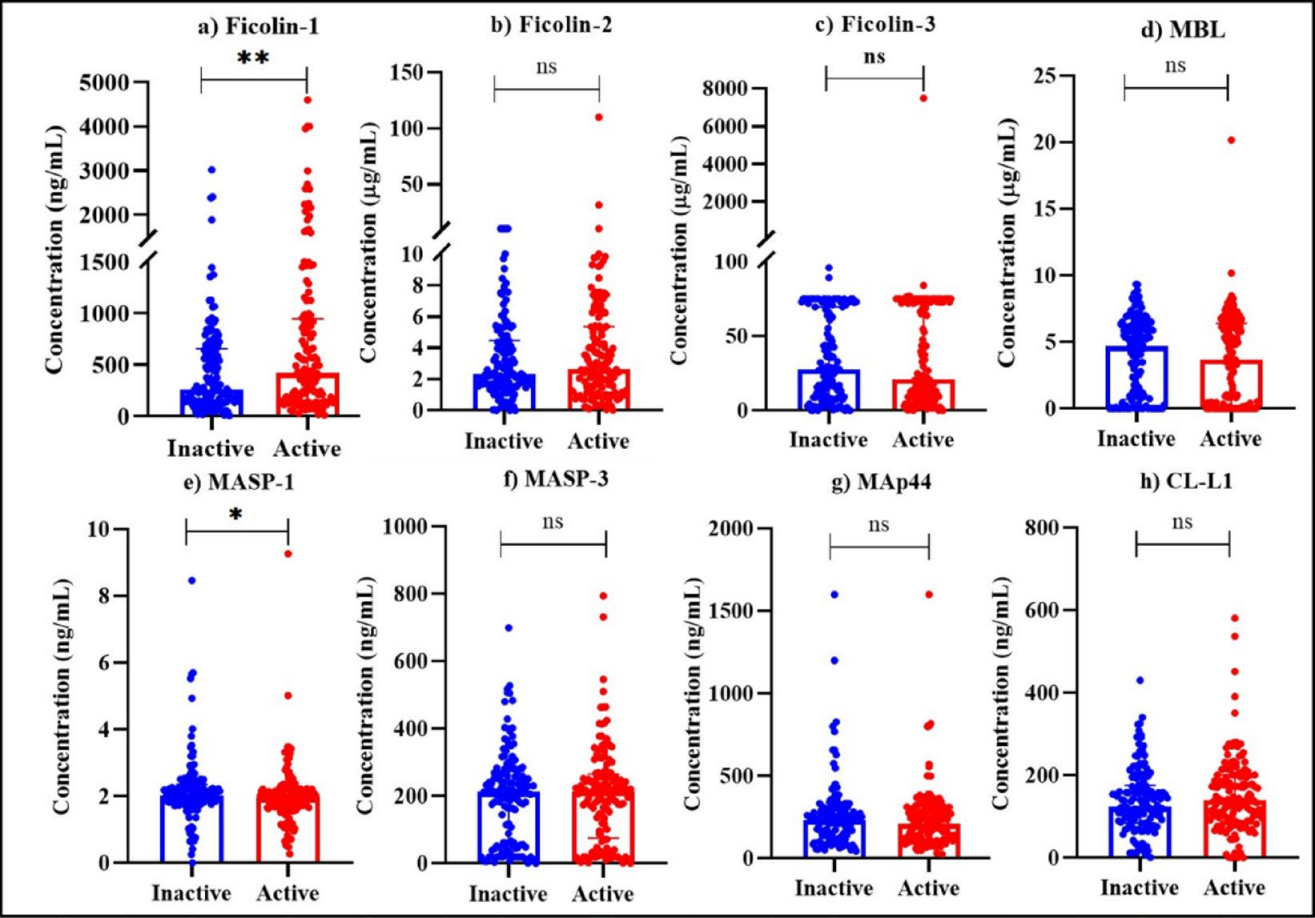
Comparison of serum lectin pathway PRMs between inactive (SLEDAI < 7; *n* = 138) and active (SLEDAI ≥ 7; *n* = 144) SLE patients. Serum levels of (**a**) ficolin-1, (**b**) ficolin-2, (**c**) ficolin-3, (**d**) MBL, (**e**) MASP-1, (**f**) MASP-3, (**g**) MAp44 and (**h**) CL-L1 were measured using ELISA. Data are presented as individual values (dots), with bars representing the medians. Group comparisons were performed using the Mann-Whitney U test. Statistical significance is indicated as follows: *p < 0.05, **p < 0.01, ***p < 0.001.

Variation in lectin pathway-associated PRMs with respect to disease activity was assessed in active and inactive SLE patients. In comparison to inactive SLE patients, active SLE patients had significantly elevated ficolin-1 (*p = 0.009*) levels (Fig. 2 (a)). Whereas, active SLE patients had significantly reduced MASP-1 (*p = 0.022*) levels in comparison to inactive SLE patients(Fig. 2 (e)). No significant differences were observed in ficolin-2, ficolin-3, MBL, MASP-3, MAp44 and CL-L1 levels (Fig. 2 (b), (c), (d), (f), (g), and (h)).

### Association of lectin pathway-associated PRMs with clinical manifestations in SLE patients

The association between lectin pathway-associated PRMs and clinical manifestations among SLE patients is summarized in Table 2. SLE patients with renal involvement had elevated ficolin-1 (*p = 0.009*) levels, while those with hematological manifestations had a significantly reduced MASP-1 (*p = 0.018*), MASP-3 (*p = 0.002*), and MAp44 (*p = 0.002*) levels. SLE patients with mucocutaneous manifestations had significantly elevated MAp44 (*p < 0.001*) levels, while those with constitutional manifestations had significantly reduced MAp44 (*p = 0.047*) levels.

**Table 2:** Association of lectin pathway-associated PRMs with clinical manifestations in SLE patients.

Clinical Manifestations		Ficolin-1 (ng/mL)	*p*-value	Ficolin-2 (μg/mL)	*p*-value	Ficolin-3 (μg/mL)	*p*-value	MBL (μg/mL)	*p*-value	MASP-1 (ng/mL)	*p*-value	MASP-3 (ng/mL)	*p*-value	MAp44 (ng/mL)	*p*-value	CL-L1 (pg/mL)	*p*-value
Renal involvement	Absent (*n*=170)	264.60 (129.92, 690.72)	***0.009***	2.38 (1.28, 4.32)	*0.193*	27.97 (9.61, 72.70)	*0.169*	4.28 (0.31, 6.35)	*0.864*	1.99 (1.75, 2.23)	*0.778*	206.17 (52.82, 257.34)	*0.126*	234.22 (125.00, 308.17)	*0.158*	129.76 (84.57, 176.68)	*0.123*
	Present (*n*=112)	458.97 (193.67, 871.76)		2.76 (1.48, 5.41)		18.91 (6.89, 70.75)		4.31 (0.35, 6.43)		1.98 (1.67, 2.25)		220.69 (75.50, 308.34)		203.50 (118.76, 277.07)		140.90 (94.36, 108.75)	
Hematological	Absent (*n*=186)	358.56 (137.97, 793.51)	*0.945*	2.52 (1.47, 4.39)	*0.651*	21.01 (7.18, 72.02)	*0.144*	4.20 (0.44, 6.13)	*0.948*	2.00 (1.77, 2.26)	***0.018***	218.92 (119.26, 288.71)	***0.002***	239.78 (142.07, 305.93)	***0.002***	138.33 (95.02, 193.28)	*0.604*
	Present (*n*=96)	326.63 (144.12, 725.14)		2.56 (1.16, 5.89)		28.64 (11.53, 72.76)		4.55 (0.10, 6.61)		1.92 (1.59, 2.17)		188.96 (32.17, 243.20)		171.10 (98.91, 272.73)		128.73 (81.45, 198.53)	
Mucocutaneous	Absent (*n*=226)	363.17 (144.68, 826.25)	*0.069*	2.54 (1.40, 5.34)	*0.388*	22.336 (8.49, 71.96)	*0.646*	4.20 (0.22, 6.40)	*0.759*	1.96 (1.66, 2.25)	*0.075*	211.57 (52.82, 268.98)	*0.608*	201.90 (106.25, 282.51)	***<0.001***	139.34 (84.57, 200.98)	*0.310*
	Present (*n*=56)	264.60 (119.91, 641.78)		2.47 (1.43, 3.86)		26.84 (8.06, 75.00)		4.55 (0.88, 5.97)		2.04 (1.95, 2.21)		216.29 (118.54, 266.06)		263.85 (228.14, 333.38)		126.07 (103.37, 154.03)	
Musculoskeletal	Absent (*n*=233)	332.70 (140.53, 741.39)	*0.641*	2.44 (1.44, 5.16)	*0.808*	23.44 (8.62, 72.09)	*1.000*	4.54 (0.34, 6.40)	*0.411*	1.97 (1.71, 2.26)	*0.564*	212.27 (55.22, 266.06)	*0.585*	223.08 (118.76, 298.05)	*0.760*	128.01 (84.14, 194.94)	*0.182*
	Present (*n*=49)	359.00 (143.03, 892.50)		2.61 (1.23, 4.47)		22.27 (6.92, 72.94)		3.65 (0.21, 6.20)		2.01 (1.83, 2.22)		217.19 (112.94, 286.47)		235.90 (124.22, 304.96)		149.11 (101.95, 190.77)	
Constitutional	Absent (*n*=171)	352.00 (143.20, 801.45)	*0.985*	2.31 (1.34, 4.37)	*0.077*	20.03 (7.21, 69.70)	*0.094*	4.59 (0.41, 6.38)	*0.697*	1.99 (1.77, 2.23)	*0.348*	209.95 (52.17, 280.54)	*0.964*	235.90 (126.60, 307.69)	***0.047***	125.63 (79.83, 187.82)	*0.292*
	Present (*n*=111)	307.51 (138.18, 764.00)		2.93 (1.64, 5.42)		28.12 (9.44, 73.12)		4.05 (0.14, 6.39)		1.96 (1.60, 2.29)		215.94 (119.62, 257.92)		206.41 (106.30, 278.72)		142.77 (95.16, 198.60)	

Data are presented as median (Q_1_, Q_3_). Comparisons between groups with and without specific clinical manifestations were performed using the Mann–Whitney U test. *p*<0.05 was considered statistically significant.

### Serum levels of autoantibodies against Ficolins and MBL in SLE patients in comparison to HCs

**Fig. 3:**
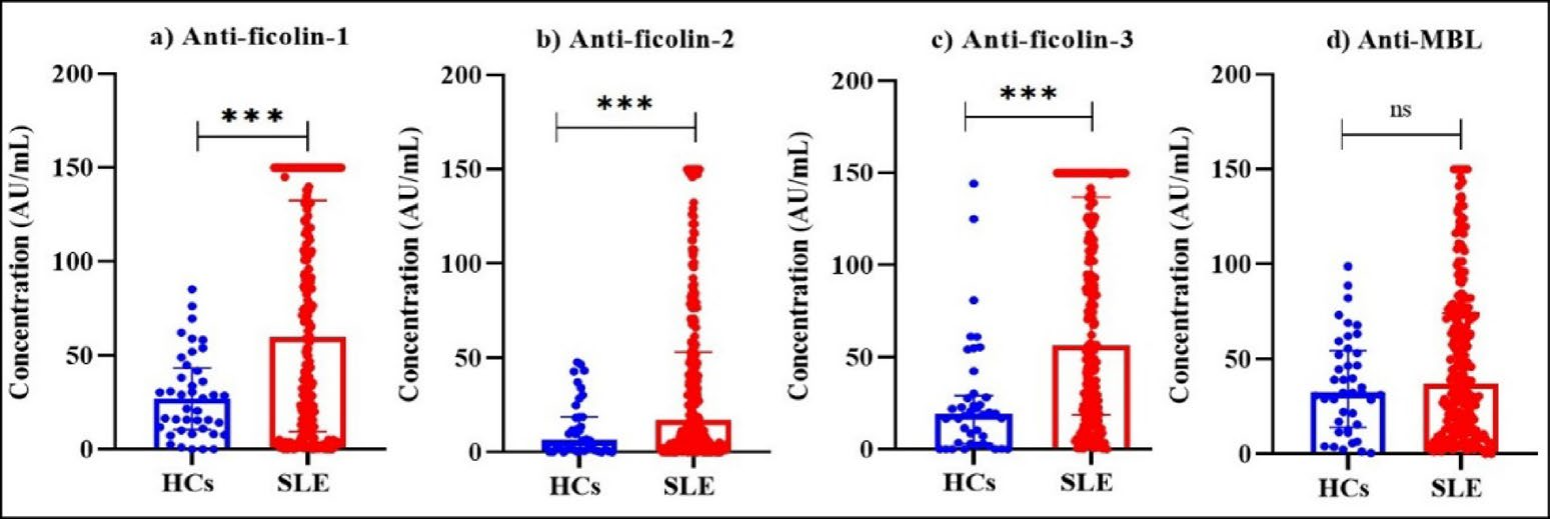
Comparison of autoantibodies against ficolins and MBL between HCs (*n* = 40) and SLE patients (*n* = 250). Serum levels of (**a**) anti-ficolin-1, (**b**) anti-ficolin-2, and (**c**) anti-ficolin-3 autoantibodies and (**d**) anti-MBL antibodies were measured using ELISA. Data are presented as individual values (dots), with bars representing the medians. Group comparisons were performed using the Mann-Whitney U test. Statistical significance is indicated as follows: *p < 0.05, **p < 0.01, ***p < 0.001.

Alterations in the anti-ficolin autoantibodies and anti-MBL antibodies were investigated by comparing their serum levels among SLE patients and HCs. In comparison to HCs, SLE patients had significantly elevated anti-ficolin-1 (*p = 0.001*), anti-ficolin-2 (*p = 0.001*), and anti-ficolin-3 (*p < 0.001*) autoantibodies (Fig. 3 (a–c)). No significant difference was observed in anti-MBL antibody levels (Fig. 3**(d)**).

### Association of anti-ficolin autoantibodies and anti-MBL antibodies with clinical manifestations in SLE patients

The association of anti-ficolin-1, anti-ficolin-2, anti-ficolin-3, and anti-MBL antibodies with clinical manifestations is summarized in Table 3. SLE patients with mucocutaneous manifestations had significantly elevated anti-ficolin-1 (*p = 0.038*) autoantibodies, while those with musculoskeletal manifestations had reduced anti-ficolin-3 (*p = 0.007*) autoantibodies.

**Table 3:** Association of anti-ficolin autoantibodies and anti-MBL antibodies with clinical manifestations in SLE patients.

		Anti-ficolin-1 (AU/mL)	*p*-value	Anti-ficolin-2 (AU/mL)	*p*-value	Anti-ficolin-3 (AU/mL)	*p*-value	Anti-MBL (AU/mL)	*p*-value
Renal involvement	Absent (*n*=170)	67.62 (16.02, 141.22)	*0.097*	19.23 (5.15, 52.89)	*0.382*	59.97 (20.52, 136.94)	*0.433*	29.81 (11.86, 68.93)	*0.105*
	Present (*n*=112)	38.47 (3.40, 129.70)		15.55 (1.85, 58.05)		47.77 (16.36, 139.44)		45.71 (11.63, 78.02)	
Hematological	Absent (*n*=186)	52.06 (9.78, 130.37)	*0.463*	18.14 (3.55, 50.21)	*0.934*	55.80 (18.32, 125.01)	*0.304*	38.91 (12.82, 74.80)	*0.402*
	Present (*n*=96)	68.12 (7.01, 141.58)		15.98 (3.44, 62.41)		75.14 (18.70, 150.00)		32.31 (9.57, 72.91)	
Mucocutaneous	Absent (*n*=226)	50.01 (7.43, 121.99)	***0.038***	15.55 (3.32, 49.51)	*0.372*	53.68 (16.44, 133.96)	*0.093*	38.42 (11.59, 74.70)	*0.665*
	Present (*n*=56)	91.75 (15.50, 150.00)		27.25 (3.99, 76.72)		73.12 (30.66, 150.00)		27.12 (11.74, 68.40)	
Musculoskeletal	Absent (*n*=233)	58.75 (6.45, 131.72)	*0.398*	18.59 (3.07, 57.47)	*0.764*	73.12 (23.64, 143.80)	***0.007***	35.61 (11.54, 72.73)	*0.168*
	Present (*n*=49)	64.01 (19.33, 147.48)		15.77 (4.94, 47.70)		24.71 (8.91, 108.20)		55.07 (12.66, 89.91)	
Constitutional	Absent (*n*=171)	53.85 (5.38, 139.47)	*0.260*	13.99 (2.02, 56.23)	*0.155*	56.29 (19.32, 138.55)	*0.974*	38.39 (11.00, 72.65)	*0.693*
	Present (*n*=111)	66.63 (19.80, 130.71)		24.84 (8.20, 46.60)		56.97 (18.43, 126.22)		35.68 (14.72, 74.73)	

Data are presented as median (Q_1_, Q_3_). Comparisons between groups with and without specific clinical manifestations were performed using the Mann–Whitney U test. *p*<0.05 was considered statistically significant.

### Correlation between lectin pathway-associated prms, SLEDAI, and other laboratory parameters in SLE patients

**Fig. 4:**
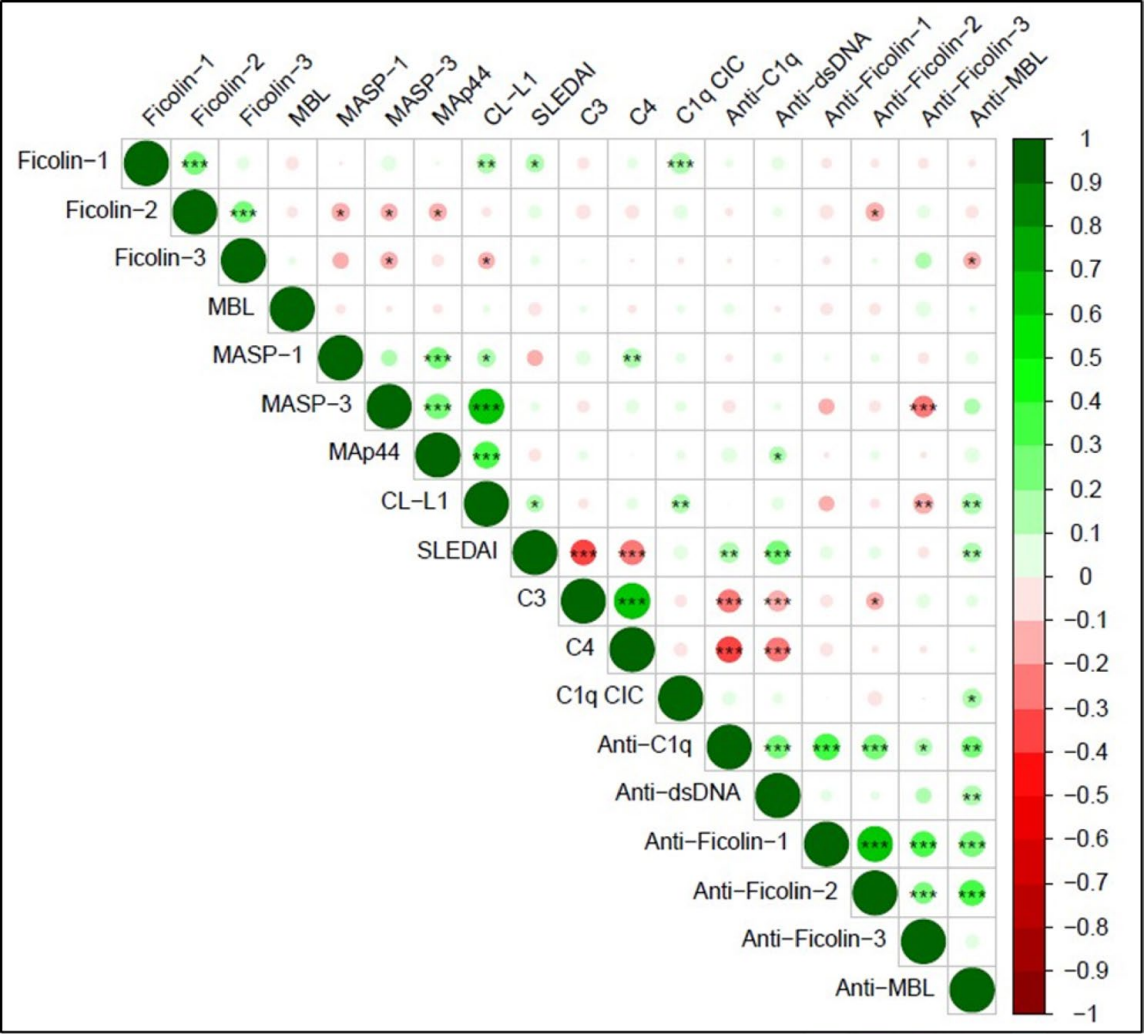
Correlation matrix showing associations among lectin pathway PRMs, their corresponding autoantibodies, complement components, and disease activity in SLE patients. Spearman’s rank correlation coefficients were calculated and Spearman’s rank correlation tests were performed. The color scale indicates the strength and direction of correlation (green: positive; red: negative), and the size of the circles corresponds to the magnitude of correlation. Statistical significance is indicated as follows: *p < 0.05, **p < 0.01, ***p < 0.001.

The association between lectin pathway-associated PRMs, SLEDAI, and other laboratory parameters was assessed using Spearman’s rank correlation analysis (Fig. 4). The SLEDAI score had a positive correlation with ficolin-1 (*r* = 0.144, p = 0.016) and CL-L1 (*r* = 0.126, *p* = 0.035) levels, while a negative correlation with C3 (*r*=-0.035, *p* < 0.001) and C4 (*r*=-0.284, *p* < 0.001) levels. Ficolin-1 levels had a positive correlation with ficolin-2 (*r* = 0.232, *p* < 0.001), CL-L1 (*r* = 0.170, *p* = 0.004) and C1q CIC (*r* = 0.197, *p* = 0.001) levels. Ficolin-2 levels had a positive correlation with ficolin-3 (*r* = 0.208, *p* < 0.001), whereas a negative correlation with MASP-1 (*r*=-0.146, *p* = 0.014), MASP-3 (*r*=-0.124, *p* = 0.037) and MAp44 (*r*=-0.135, *p* = 0.024) levels. Ficolin-3 levels had a negative correlation with MASP-3 (*r*=-0.130, *p* = 0.030) and CL-L1 (*r*=-0.121, *p* = 0.042) levels. MASP-1 levels had a positive correlation with MAp44 (*r* = 0.204, *p* = 0.001), CL-L1 (*r* = 0.147, *p* = 0.014) and C4 (*r* = 0.164, *p* = 0.006) levels. MASP-3 levels had a positive correlation with MAp44 (*r* = 0.293, *p* < 0.001) and CL-L1 (*r* = 0.612, *p* < 0.001) levels. MAp44 levels had a positive correlation with CL-L1 (*r* = 0.332, *p* < 0.001) and anti-dsDNA antibody (*r* = 0.124, *p* = 0.038) levels. CL-L1 levels had a positive correlation with C1q CIC (*r* = 0.156, *p* = 0.009) levels.

### Correlation of anti-ficolin autoantibodies and anti-MBL antibodies with disease activity and other laboratory parameters

Spearman’s rank correlation analysis was employed to assess the correlation between anti-ficolin autoantibodies and anti-MBL antibodies with SLEDAI, and other laboratory parameters (Fig. 4). The SLEDAI score had a positively correlation with anti-C1q (*r* = 0.190, *p* = 0.001), anti-dsDNA (*r* = 0.265, *p* < 0.001) and anti-MBL (*r* = 0.169, *p* = 0.007) antibodies. Anti-ficolin-1 autoantibodies had a positive correlation with anti-C1q (*r* = 0.329; *p* < 0.001), anti-ficolin-2 (*r* = 0.620; *p* < 0.001), anti-ficolin-3 (*r* = 0.304; *p* < 0.001) and anti-MBL (*r* = 0.296; *p* < 0.001) antibodies. Anti-ficolin-2 levels had a positive correlation with anti-C1q (*r* = 0.285; *p* < 0.001), anti-ficolin-3 (*r* = 0.208; *p* = 0.001) and anti-MBL antibodies (*r* = 0.302; *p* < 0.001), whereas a negative correlation with ficolin-2 (*r*=-0.153, *p* = 0.015) and C3 (*r*=-0.131; *p* = 0.039) levels. Anti-ficolin-3 autoantibodies had a positive correlation with anti-C1q (*r* = 0.131; *p* = 0.039) autoantibodies, whereas a negative correlation with MASP-3 (*r*=-0.211; *p* = 0.001) and CL-L1 (*r*=-0.187; *p* = 0.003) levels. Anti-MBL antibodies had a positive correlation with anti-C1q (*r* = 0.204; *p* = 0.001), anti-dsDNA antibodies (*r* = 0.178, *p* = 0.005), CL-L1 (*r* = 0.197; *p* = 0.002) and C1q CIC (*r* = 0.160; *p* = 0.011) levels, whereas a negative correlation with ficolin-3 (*r*=-0.127, *p* = 0.046) levels.

## Discussion

SLE, a systemic prototype autoimmune disease results from dysregulation of both innate and adaptive immunity, leading to inflammation and/or tissue/organ damage. Though the role of pathogenic autoantibodies and immune complexes in SLE is well defined, recent studies emphasize an essential role of the innate immunity, with the complement as a central player. Dysregulation of the complement pathway results in hypocomplementemia, which is a hallmark feature observed among SLE patients. The lectin pathway is one of the three complement activation pathways that plays an important role in immune surveillance, pathogen recognition, and clearance of apoptotic debris. However, its role in SLE remains poorly characterized^14^.

In the present study, ficolin-1 levels were comparable between SLE patients and HCs. However, active SLE patients demonstrated significantly elevated ficolin-1 levels than inactive SLE patients, with a positive association with SLEDAI score, suggesting their potential role as an immunological indicator of active disease. Additionally, ficolin-1 levels were associated with renal involvement. These findings were similar to reported by Hein et al., where ficolin-1 levels were comparable between Danish SLE patients and HCs^6^. In contrast, a study conducted by Troldborg et al. reported that Danish SLE patients had significantly reduced ficolin-1 levels in comparison to HCs, with no association with SLEDAI, anti-dsDNA antibodies, and C3 levels^7^. A similar study by Troldborg et al. reported significantly reduced ficolin-1 levels in Danish SLE patients with a negative correlation with SLEDAI and a positive correlation with C3 levels^15^. Furthermore, a study by Tanha et al. in Danish SLE patients reported elevated ficolin-1 levels which were associated with the histological subtypes of LN^16^, suggesting their role in renal pathology. These findings suggest that ficolin-1 may play a role in glomerular inflammation in lupus nephritis (LN) and could serve as an immunological indicator of renal involvement. Further studies, including histopathological assessments, are warranted to confirm its specificity and temporal association with disease activity or renal flares.

In the present study, although ficolin-2 levels were significantly elevated in SLE patients in comparison to HCs, no correlation was observed between these levels and the SLEDAI score. Similar findings have been reported in studies from Denmark by Troldborg et al. and Hein et al., where elevated ficolin-2 levels did nxot show any significant association with disease activity or other laboratory parameters^6,7,15^. Conversely, a study by Watanabe et al. in the Japanese population reported that ficolin-2 levels were significantly reduced in SLE patients in comparison to HCs, with no associations with clinical manifestations and laboratory parameters. Also, ficolin-2 levels were comparable between inactive and active SLE patients^17^. In the present study, ficolin-3 levels were comparable between SLE patients and HCs and there was no correlation with SLEDAI score. This contrasts with earlier studies reporting elevated ficolin-3 levels in SLE patients in comparison to HCs^6,7,15,18^. The variations in these findings could be attributed to the differences in genetic background, environmental exposures, disease severity, treatment regimen, and method used for detection.

There are contradictory results from various populations about MBL’s involvement in the pathophysiology of SLE. Evidence suggest that MBL may have a dual role in the pathogenesis of SLE. Elevated MBL levels enhance complement activation and contribute to tissue injury, whereas MBL reduction /deficiency is associated with impaired clearance of apoptotic cells, potentially driving autoantibody production^19,20^. In the present study, SLE patients had significantly reduced antigenic MBL levels in comparison to HCs, suggesting a possible role of MBL reduction/ deficiency in impaired clearance of apoptotic cells and immune dysregulation in SLE, potentially contributing to the loss of self-tolerance and disease development. However, a study from the Eastern Indian population had reported significantly elevated antigenic MBL levels with a positive correlation with SLEDAI, anti-dsDNA antibodies, and a negative correlation with C3 and C4 levels^19^. Other studies, however, had reported comparable levels of antigenic MBL among SLE patients and HCs^7,15,21–23^. Ethnic and genetic differences, environmental exposures, heterogeneity in disease activity, or methodological variations could account for these discrepancies in findings. Antigenic MBL levels are also known to be influenced by genetic polymorphisms in the *MBL2* gene, which vary among populations^21–27^. These genetic differences may partially explain the inconsistencies in circulating antigenic MBL levels and their associations with disease activity across studies.

Levels of MASP-1 were significantly reduced in SLE patients in comparison to HCs in the present study. These findings are in line with the study by Asanuma et al., who observed reduced MASP-1 levels in both proliferative LN and non-LN SLE patients in comparison to HCs^28^. Further, the observation that active SLE patients demonstrated significantly reduced MASP-1 levels in comparison to inactive SLE patients suggests their consumption during the disease course, similar to complement consumption, thereby supporting their potential role in SLE pathogenesis. In a longitudinal study, Troldborg et al. reported comparable levels of MASP-1 in SLE patients in comparison to HCs^15^. This contrasts with their earlier study in 2015, where they found significantly elevated MASP-1 levels in patients with SLE in comparison to HCs^29^. In the present study, SLE patients had significantly elevated levels of MASP-3 and MAp44 in comparison to HCs. These findings are consistent with a study by Troldborg et al.^15,29^. This suggests a possible upregulation of certain lectin pathway PRMs, potentially reflecting a compensatory mechanism or differential regulation within the pathway. The levels of CL-L1 were comparable between SLE patients and HCs in the present study. In contrast, a study by Troldborg et al. reported that SLE patients had significantly reduced CL-L1 levels^7,15^. The observed reduction in MASP-1, MASP-3, and MAp44 levels in patients with SLE with hematological manifestations suggested their potential involvement in immune-mediated cytopenias. Further, a statistically significant association among lectin pathway-associated PRMs suggested their coordinated regulatory role in complement activation in SLE.

Autoantibodies play a crucial role in the development and progression of SLE. While autoantibodies against lectin pathway-associated PRMs have been identified, these autoantibodies have been less extensively studied in SLE, and their significance remains unclear. These autoantibodies may modulate the complement activation, influencing the pathogenesis of SLE. In the present study, significantly elevated levels of autoantibodies against ficolin-1, ficolin-2, and ficolin-3 were observed in SLE patients in comparison to HCs. These findings align with previous reports demonstrating elevated anti-ficolin-2 and anti-ficolin-3 autoantibodies in patients with SLE^4,5,30^. The significantly elevated anti-ficolin-1 autoantibodies among SLE patients with mucocutaneous involvement suggested a potential role of these autoantibodies in skin-related pathology.

The observed positive correlation between anti-ficolin-1, anti-ficolin-2, anti-ficolin-3 autoantibodies, and anti-MBL antibodies with anti-C1q autoantibodies suggested a link between the lectin and classical complement pathways, which eventually leads to complement activation. The anti-ficolin-2 autoantibodies had a negative correlation with C3 levels, suggesting that anti-ficolin-2 autoantibodies may support complement consumption, potentially leading to hypocomplementemia, a common feature of active SLE. A negative correlation was observed between anti-ficolin-2 autoantibodies with ficolin-2 levels, consistent with findings previously reported by Colliard et al., 2018^5^. This suggested that anti-ficolin-2 autoantibodies may form immune complexes with ficolin-2, which may get deposited in various tissues resulting in inflammation and tissue injury. The anti-MBL antibodies were comparable between SLE patients and HCs, however, their levels had a positive correlation with SLEDAI and anti-dsDNA antibodies. These findings suggest that anti-MBL antibodies may influence disease activity and can possibly be used as a potential immunological biomarker for assessing disease activity in SLE. How the lectin pathway contribute to SLE disease pathogenesis is not fully understood. The present study gave an insight in this regard. As per the available literature, this is the first study that comprehensively assessed the lectin pathway-associated PRMs and their corresponding autoantibodies in Indian SLE patients. Present study revealed significantly altered levels of lectin pathway PRMs (including ficolin-2, MBL, MASP-1, MASP-3, and MAp44), as well as increased levels of their corresponding autoantibodies in SLE patients compared to HCs. Further, specific PRMs and autoantibodies showed significant associations with clinical manifestations, disease activity scores (SLEDAI), and complement-related parameters (C3, C4, C1q-CIC, and anti-C1q), which are known indicators of complement activation and inflammation. These observations supported the hypothesis that the lectin pathway play a role in modulating complement activation and inflammatory responses in SLE, and point towards its potential role in contributing to disease pathogenesis. However, given that only antigenic protein levels were assessed, functional assays and mechanistic studies were required to establish causality and to elucidate the precise role of these molecules in SLE pathogenesis. This cross-sectional study provided single time point information, limiting the ability to assess the dynamic changes in the lectin pathway-associated PRMs and associated autoantibodies over the disease course. Longitudinal studies in this regard will help in better understanding the treatment response. The present study from Western India lacks the information about ethnically different geographic locations across the country to evaluate differences in lectin pathway-associated PRMs and associated autoantibodies among various ethnic backgrounds. 

In conclusion, the present study offered an insight into the altered serum levels of lectin pathway-associated PRMs and their corresponding autoantibodies among SLE patients from Western India. Significant alterations in the levels of ficolin-2, antigenic MBL, MASPs, and MAp44 levels, along with anti-ficolin autoantibodies, and their associations with clinical manifestations, disease activity and complement-related parameters suggested their potential utility as biomarkers in SLE. Further, mechanistic and longitudinal studies are warranted to validate these findings and establish causal relationships.

## Data Availability

The datasets generated during and/or analysed during the current study are available from the corresponding author on reasonable request.
